# Key factors for an equitable health intervention for Black men with low income and chronic illness in a southern state in the United States

**DOI:** 10.1371/journal.pone.0358797

**Published:** 2026-09-22

**Authors:** Sarah Alexandra Marshall, O’Dell Johnson, George Pro, Ashley Williams, Nickolas Zaller, Brooke E. E. Montgomery

**Affiliations:** 1 Department of Health Behavior & Health Education, Fay W. Boozman College of Public Health, University of Arkansas for Medical Sciences, Little Rock, Arkansas, United States of America; 2 College of Nursing, University of Arkansas for Medical Sciences, Little Rock, Arkansas, United States of America; New York City Department of Health and Mental Hygiene, UNITED STATES OF AMERICA

## Abstract

Black men’s health continues to be worse than that of many other groups in the United States. This study’s purpose was to explore interest holders’ perceptions about an intervention proposed to use universal basic income (UBI) to facilitate healthcare access and promote health equity of Black men with chronic conditions, including HIV, and low incomes in a southern state. Semi-structured interviews with 31 interest holders, including members of the priority population, were conducted via phone or Zoom. Pre-implementation factors were identified using the Socioecological Model as a framework for conventional content analysis. Participants noted that certain factors must be considered for any intervention to be successful in improving the health status of the priority population. The following themes were identified: 1) The priority population typically delays healthcare. 2) Relationships are strained due to incarceration or poverty. 3) Correctional health and healthcare systems are not structured to help. 4) Many barriers to health/healthcare exist within the community. 5) Racism and poverty are often intertwined and are entrenched barriers to health. Equitable interventions, such as providing a UBI, which are designed to improve the health and well-being of the priority population, should address the identified factors at the individual, interpersonal, community, and institutional levels to be effective. Societal factors, such as structural racism and generational poverty, may be too difficult to address in such an intervention.

## Introduction

Black men in the United States face persistent and significant health disparities, exhibiting worse outcomes than nearly every other demographic group. Compared to White men, Black men have higher rates of chronic illness, lower life expectancy, and disproportionate exposure to adverse social determinants of health [1]. Black men in the U.S. are more likely to have higher rates of hypertension [2], type II diabetes [2,3], heart disease [2,3], prostate cancer [3], and HIV [4], compared to White men. Despite greater disease burden, historically Black men report lower use of ambulatory healthcare services than White men [5]. Economically, Black men earn only 63.6% of what White men earn, are more than twice as likely to live below the poverty line, and nearly five times more likely to be incarcerated [6]. These disparities are not simply individual or behavioral but are deeply rooted in structural racism and discrimination (SRD) [7,8].

SRD refers to systemic policies, institutional practices, and societal norms that produce and reinforce racial inequities across domains—healthcare access, employment, housing, and criminal justice [9]. For Black men with low incomes and chronic conditions, SRD often manifests in limited access to quality healthcare, food insecurity, over-policing, environmental hazards, residential segregation, and heightened vulnerability to incarceration [10]. These interlocking disadvantages disrupt basic human needs—such as safety, housing, and community—obstructing opportunities for self-actualization and long-term health [7].

The mass incarceration of Black men is a clear consequence of SRD, with long-term health and social impacts. Though the 2018 First Step Act expanded rehabilitative and reentry programs for justice-impacted (JI) individuals in federal systems, reentry challenges remain substantial [11]. Black men often return to communities facing over 430 collateral consequences that limit access to employment, healthcare, housing, and social services—factors critical for stability and health equity [12]. Additionally, a history of incarceration has well-documented deleterious impacts on both health status and health-related outcomes, particularly among Black men who have historically been disproportionately impacted by mass incarceration in the United States [13,14]. Health sequelae often persist long after release from incarceration [15,16] and are exacerbated by economic and related insecurities [17].

In response to these compounded inequities, there is an urgent need for interventions that address not only clinical needs but also broader structural barriers to health and healthcare access. This study sought to identify interest holders’ perspectives on universal basic income (UBI) [18] –also known as guaranteed supplemental income—as a potential intervention to improve healthcare access and promote health equity for Black men with low incomes and chronic illnesses [19–21]. While previous studies have examined the effects of unconditional cash transfers with various individuals with low incomes in the U.S., including those formerly incarcerated [22], few have examined the multilevel factors that members of the priority population as well as the community-based partners and service providers that serve them consider essential before implementing UBI intervention so that adoption, implementation, and sustainability are maximized. The goal of this manuscript is not to describe why UBI is needed nor to describe the intervention’s design. These are related yet different questions. Instead, we aim to examine factors across the Social Ecological Model that must be accounted for during the pre-implementation phase for a UBI intervention to be successful in real-world settings among Black men with chronic illness. Interest holder input, particularly from those in service delivery, regulation, and advocacy, is essential for developing effective, community-based interventions. Understanding their views may inform future health policy and practice aimed at advancing health justice for this priority population.

## Materials and methods

This study followed the consolidated criteria for reporting qualitative research (COREQ) checklist. Members of the research team collaborated with existing contacts and new referrals in the healthcare system, criminal justice system, and community-based organizations that typically serve the priority population—i.e., Black men with low incomes (defined as living 11.5% below the poverty line) and chronic health conditions who are not engaged in healthcare and possible involvement in the criminal justice system; and representatives of the priority population themselves were also invited to participate. The University of Arkansas for Medical Sciences’ Institutional Review Board (IRB) approved the study. Information about the study was provided in flyers and email communications to recruit interview participants within the systems or organizations of interest. Individual interviews were conducted with the following categories of purposively selected interest holders: 1) healthcare system workers, 2) criminal justice professionals, 3) community leaders, 4) HIV-related community leaders, and 5) members of the priority population (i.e., Black men with low incomes, chronic health conditions, not engaged in healthcare, and possible involvement in the criminal justice system). Of note, HIV-related interest holders were added to the study sample through supplemental funding to the parent grant to explore the potential benefits of a UBI on engaging out of care Black men living with HIV into HIV care.

Members of the research team conducted the semi-structured interviews between November 2022 and January 2023 with an interview guide that explored perceptions about providing UBI to Black men with low incomes and chronic illness and that gathered information about culturally acceptable methods through which to implement a future UBI intervention. Interview participants were asked about the factors that may influence the overall health and well-being and the healthcare-use decisions of low-income Black men. Participants were told that this information would be used to inform the development and testing of a UBI intervention focused on increasing healthcare use among low-income Black men living with a chronic disease who were not engaged in healthcare. Interviews were scheduled for 60–90 minutes, depending on the amount of time the participant was willing to give, and were conducted via telephone or Zoom; all were audio recorded. Each participant verbally consented before beginning the interview. Written consent was not obtained to protect participant anonymity, and the university’s IRB approved the use of verbal consent, which was recorded by the interviewee. Because the interviews were conducted via telephone or Zoom, participants were able to choose a location that allowed privacy. No identifying information was reported in the interview findings, and any individual identifiers have been removed or changed in the presentation of the results. Each interview participant was given a check for $50 after completing the interview.

A professional transcription service transcribed the audio recording of each interview. Transcribed data were uploaded into MAXQDA22, a qualitative data analysis package, and members of the research team used conventional methods for content analysis [23]. The research team used the interview guide to deductively derive the initial codebook. The researchers who conducted the interviews independently coded the deidentified interview transcripts; multiple coders enhanced the rigor of the analysis [24]. The group met multiple times in the spring of 2023 to discuss their understanding and application of the coding definitions in the preliminary codebook; discrepancies in coding were resolved, and consensus was achieved for revising coding definitions and adding emergent codes.

**Fig 1:**
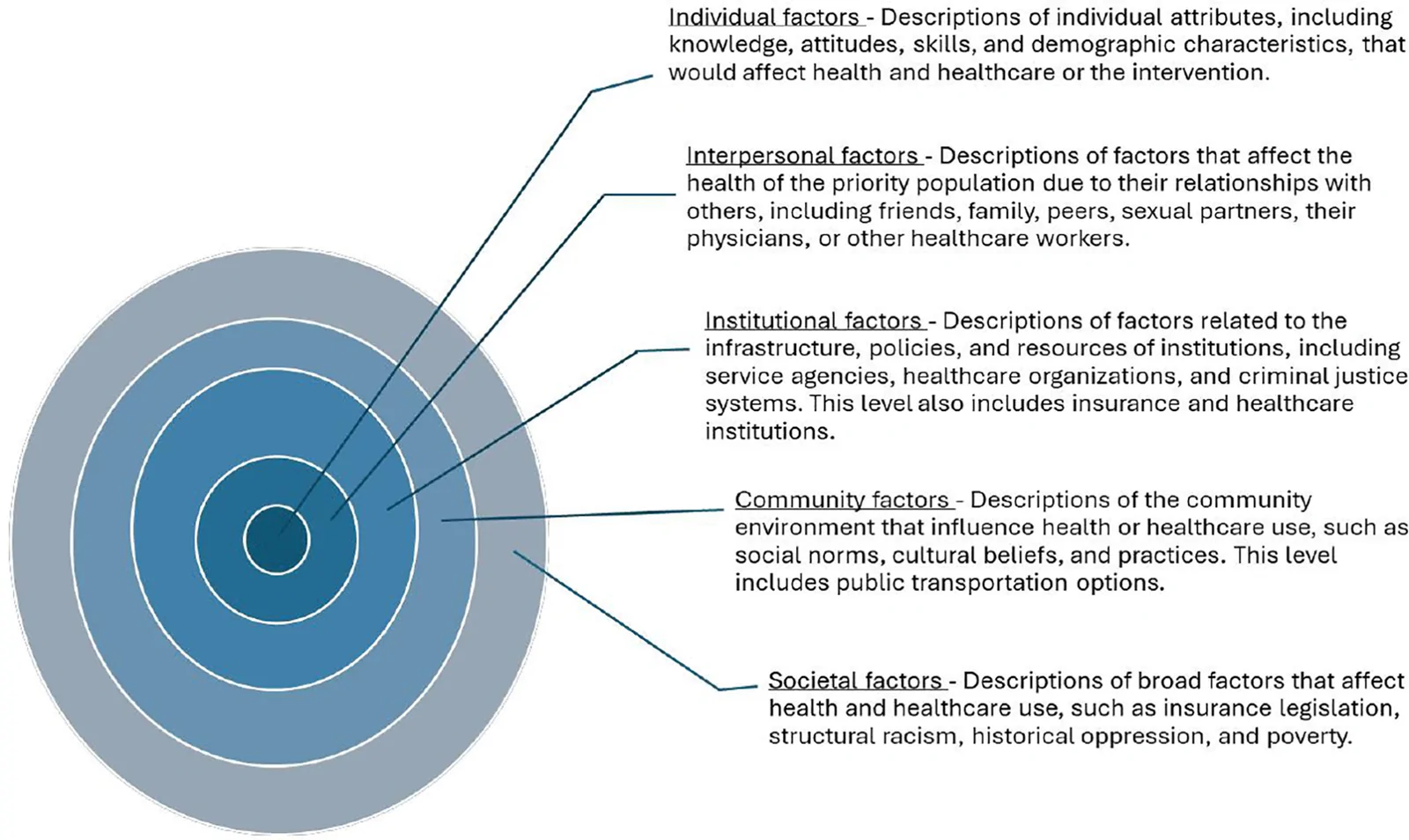
Socioecological Model with Coding Definitions.

Coding was completed in two phases. In phase 1, segments of transcripts were coded with “pre-implementation” in the summer of 2023 if the participant described factors that may affect the perceived need, acceptability, and feasibility of a UBI intervention or other health-improvement efforts. This included responses relevant to factors that affect healthcare, personal health, or a UBI intervention. In phase 2, the segments already coded as “pre-implementation” were further categorized and coded according to the factors of the socioecological model (SEM) (i.e., individual factors, interpersonal factors, institutional factors, community factors, and societal factors) in the fall of 2023. Coding definitions for each of the SEM factors are shown in Fig 1.

One researcher used MAXQDA22 for a complex coding query that pulled text segments dually coded as “pre-implementation” and each of the five SEM factors. This process resulted in five dually-coded text documents that contained each of the five sets of complex coding queries (e.g., pre-implementation + individual factors). From each of the five sets of complex coding queries, themes emerged that were discussed and articulated by the research team.

### Theoretical framework

The socioecological model (SEM) is a valuable framework for qualitative research in health because it helps researchers understand the complex interplay of factors that influence health behaviors and outcomes [25]. Thus, the SEM has been used in previous studies examining chronic disease interventions [26]. By considering individual, interpersonal, institutional, community, and policy levels, the SEM provides a holistic perspective that is well-suited for exploring qualitative data and understanding the lived experiences of individuals and communities [27]. The SEM provides a framework for analysis by considering the different levels of influence that contribute to a health issue or behavior [25,27,28]. Qualitative methods are particularly useful for exploring the contextual factors highlighted by the SEM. The SEM emphasizes the reciprocal relationship between individuals and their environments, allowing researchers to examine how different factors interact to influence health behaviors and outcomes. By identifying the specific levels and factors that influence health behaviors, the SEM can inform the development of effective interventions, including UBI, that address the root causes of health disparities. Assessing how some ecological levels affect subgroups differently is a critical component of health disparities measurement [29]. Applying the SEM to studies of UBI will lead to more thorough investigation of disparities, for example, how the conditions of the lives of individuals with a history of incarceration have led to the need for supplemental income in the first place. UBI is thought to provide a structural policy change that enables positive cascading effects across the multiple nested levels of influence of the SEM on the health disparities experienced by communities [30].

## Results

A total of 31 interest holders were interviewed, including healthcare providers (n = 6), individuals in the criminal justice system (n = 6), community leaders (n = 7), HIV-related advocates (n = 5), and members of the priority population (n = 7) (i.e., Black men with chronic conditions, including HIV, no healthcare engagement, low incomes and possible justice involvement). See Table 1 for characteristics of interview participants. Analysis revealed five key themes, using the SEM as a theoretical framework.

**Table 1:** Characteristics of individual interview participants.

Participant Number	Interest holder Category	Gender*	Race**
1	Criminal Justice	F	B
2	Criminal Justice	M	B
3	Criminal Justice	M	B
4	Criminal Justice	F	W
5	Criminal Justice	F	W
6	Criminal Justice	M	W
7	Community Leader	M	B
8	Community Leader	F	B
9	Community Leader	M	B
10	Community Leader	F	B
11	Community Leader	F	B
12	Community Leader	M	B
13	Community Leader	F	B
14	HIV Community Leader	M	B
15	HIV Community Leader	F	B
16	HIV Community Leader	M	B
17	HIV Community Leader	F	B
18	HIV Community Leader	M	W
19	Health Care	F	South Asian
20	Health Care	F	W
21	Health Care	M	B
22	Health Care	F	W
23	Health Care	F	B
24	Health Care	F	W
25	Priority Population	M	B
26	Priority Population	M	B
27	Priority Population	M	B
28	Priority Population	M	B
29	Priority Population	M	B
30	Priority Population	M	B
31	Priority Population	M	B

*M: male, F: female; **B: Black, W: White; None of the participants identified as Hispanic.

### Theme 1: Individual factors – delaying care due to individual experiences of trauma or hardship

The priority population has the following set of common characteristics and/or experiences: they will frequently delay care; they typically have experienced traumas or hardships; and they must have their basic needs met first. Many participants indicated that these factors must be considered when developing an intervention to improve the health and healthcare use of Black men, such as UBI.

A community leader put it plainly: “What I’ve found is that a lot of the chronically ill, low-income Black males do not go to the doctor as often as they should …unless something major happens. In other words, there’s no prevention… until an issue occurs that makes them have to go to the doctor,” (Participant #7).

A member of the priority population offered this as an explanation for why low-income Black men with a chronic condition may not seek healthcare: “…a lot of ’em just afraid to go to the doctor. They’re afraid, scared to death” (Participant #28). This participant and others referred to past injustices like the Tuskegee experiment as an underlying reason for being afraid to seek healthcare services.

Additionally, a healthcare provider who frequently provides care for this priority population offered the following as a barrier that prevents Black men from getting the healthcare they need: “Usually that’s because they had other competing challenges at home… ‘It [the patient’s health condition] doesn’t really matter to me because I need to support my family, so managing my diabetes is lower on the problem list. Until it becomes a problem that impairs my ability to care for my family.’ That’s usually what I see,” (Participant #24). The notion of competing priorities was mentioned by multiple interviewees. One participant put it this way, “I honestly think the primary thing is when you are in crisis and when you are dealing with a housing crisis, job crisis, I-might-be-going-back-to-jail crisis, everything is an emergency. Especially a long-term health issue that you’ve been dealing with for a long time, you don’t deal with that until it rises to the level of also an emergency” (Participant #5). Several participants also reported that this population routinely delays care, often because of an inability to afford it. As mentioned across several interviews, members of the priority population did not perceive their chronic health conditions as emergencies and therefore were less likely to allocate their limited resources, such as transportation, time off work, and co-pays, to attend doctor’s appointments. Their resources, including time, income, and attention, were allocated to issues and situations they perceived as true emergencies, such as housing and criminal justice system involvement. However, receipt of additional funds through UBI had the potential of improving health promoting behaviors (i.e., healthcare access) by increasing access to discretionary funds and allowing non-emergency health-related issues to be addressed. This highlights the importance of addressing the basic needs of the priority population through a UBI intervention to improve health and well-being.

### Theme 2: Interpersonal factors – otherwise supportive relationships are strained

Interpersonal relationships that might otherwise be instrumental in facilitating the health and well-being of the priority population are difficult. Family relationships often have been devastated by incarceration and/or generational poverty. Additionally, relationships with medical care providers are strained due to distrust and/or lack of a sincere connection.

A few participants mentioned that relationships could be instrumental in supporting the health and well-being of Black men. For instance, one healthcare provider said, “Many times, it is the women in their lives who notice a change and say, ‘You’ve got to get this checked out. You’ve got to go and get this looked at,’” (Participant #23). This same participant mentioned that the lack of such supportive relationships can be problematic.

The other thing, relationships are big. If they don’t have a support system, they don’t have a spouse or a girlfriend or a friendship or whatever it is, that support system can be one of the factors that stresses people because they don’t have an outlet if you will. That can also cause issues. (Participant #23)

For the priority population, crucial supportive interpersonal relationships often are negatively impacted by involvement with the criminal justice system. Considering the ways that incarceration can affect a family, one community leader offered this story:

Well, I think that all of them probably suffer from PTSD, because it’s a traumatic experience when you’re head of household, your child, your son, your father is ripped away from you and taken to jail. That can play a mental toll on children, especially. My little cousin, when my family member was incarcerated, his son was recently born. By the time he got out of jail, my little cousin was a teenager. He grew up angry. No ifs, ands, or buts about it. You could just see the anger on his face. I think that it was more sadness than anger, probably 50/50. Anger knowing that his father was incarcerated; sad knowing that his father was incarcerated. I think that weighs on the parents, the siblings, the spouses, I think all of them go through some type of mental health, some type of PTSD. Going for visits, going to the jail to visit your loved one, and knowing that in an hour or so, you have to leave to travel, the ride home, not knowing when you’re gonna be able to see your loved one again, that type of thing, that weighs heavily on a person. (Participant #10)

Another community leader summarized the effect of incarceration on a family this way:

Oh, having someone released from prison who is also a member of a household, family, children, mother, so every person in the house, first of all, they missed that loved one. When they’re back, they don’t know exactly how to treat that loved one, and the loved one don’t know exactly how to accept them again depending on how long they’ve been gone. Therefore, the family structure is broken when the person is taken out of the structure and entered prison. Now, everybody who’s gonna be in that family needs to be reallocated to one another. Roles need to be reassigned and addressed because a person coming out of prison may not know their role in the family anymore. It may have been that they were the head of the house, but they come back, they’re not the head of the house anymore ‘cause they’re not providing, and they hadn’t been providing. Everybody must be reeducated to roles and given the tools to fulfill their roles. (Participant #7)

Additionally, someone who works in the criminal justice system offered this insight:

It’s very expensive to have someone incarcerated. I don’t know if you know that. If you’re a family member. My son was incarcerated. We’re on a 1,200-calorie-a-day menu. It’s much of the same stuff every day. If people wanna supplement what they eat during the day, then they buy commissary and *[crosstalk 18:13]*. That’s an extra expense. Then you have the video chats or calls. Then you have an attorney expense. Then, many times, you have restitutions. It’s just devastating financially to a family. Plus, the loss of income. These are not people who have a fat bank account, usually. (Participant #4)

The interpersonal relationships within the environment in which Black men reside or return to may also negatively affect their health and wellbeing. A community leader said, “Of course, the environment that they live in. Again, they’re surrounded by people who may be perpetuating harmful information, or giving them things, or exposing them to things that would be harmful to their mental and physical health,” (Participant #8).

Regarding the difficulties of relationships with medical providers, one member of the priority population put it this way: “Basically, I always say it as a joke, but it’s two things I know a Black man scared of: that’s the doctor and the police.” To explain, he added, “Fear of being diagnosed with something real bad or just—to tell you the truth, just fear of history...” (Participant #30).

Another member of the priority population talked about how he wanted to be treated as an individual and not just a number. Several interview participants expressed that healthcare professionals needed to have a background or lived experiences in common with the priority population; without that commonality, these participants felt that healthcare providers would not be able to connect with Black male patients. One member of the priority population said, “Doctors don’t look like us. We don’t trust doctors. Doctors …they’re not vested in our health or in the betterment of our community,” (Participant #26).

### Theme 3: Institutional factors – correctional health and healthcare systems are not structured to help

Institutional factors like healthcare and the criminal justice system are not structured to be favorable to or supportive of low-income Black men with chronic conditions. Frequently, participants mentioned problems that affect access to healthcare, such as having insurance or being able to afford medication. Some participants talked about the need to have providers located in areas where the priority population is living or to have providers do pro bono work to provide care to low-income Black men with chronic conditions. A few interview participants, including some members of the priority population, mentioned the differential treatment they witnessed or experienced in the healthcare system. Furthermore, a few interview participants stated that the criminal justice system often created or exacerbated chronic health conditions experienced by the priority population.

A few interviewees also referred to issues with “the system.” For example, one healthcare provider characterized efforts to provide care in his healthcare institution this way:

Like we’re still requiring people to come to us and then we refer them out to resources. We kind of leave it on them to go figure out how to take advantage of those resources. Food insecurity is a perfect example. People who are food insecure. We have food boxes that we can give them, but then we say, ‘Alright, you gotta go to one of these five different pantries and try to figure it out.’ If you don’t have transportation, ‘Now, here’s the link to MetroLINK [bus system]. You’ve got to fill out this form and maybe we’ll help you, but maybe we can’t.’ There just—There’s so many additional steps that people have to take to take advantage of community resources that would address unmet social needs. We’re better about pointing people to resources for unmet social needs, but we’re not that great at closing the loop or doing—Any of those closed-loop referrals, we really don’t do. I’d say we’re okay. We could be better, but we’re better than nothing. (Participant #24)

In addition, a community leader whose brother is a Black man who has a low income and a chronic condition (and was previously incarcerated) shared this insight into an issue they encountered: “... He was needing to get dialysis and there’s transportation that can take you back and forth, but because of where we live, the transportation would not go that far and my brother didn’t have a car, so it would only take you to a certain distance, and that was it…It only provided for a certain radius,” (Participant #11).

Another community leader suggested that policy changes at the institutional level could improve the health and well-being of the priority population.

Like I said, a mechanism built into our so-called correction system, you talkin’ about [state] has the highest recidivism rate of any state. At a certain point, we were in a growth pattern that if we were an individual country, we would imprison more people than any other country in the world per capita. That’s not workin’. Why not use, like I said, the existin’ mechanism, existin’ whatever, to do somethin’ different? Because like I say, a lot of these folks by and large dealin’ with drug addiction, joblessness. They don’t have to be—I don’t think – have to be paranoid schizophrenic to need some level of mental health care. Instead of spendin’ money on piss testin’ folks and lockin’ ’em back up, we should spend it on everything from counseling to training to literally anything that makes sense for the idea which they say of keeping folks out of—from coming back to prison. They ought to keep folks healthy in general, and I think it is within the interest of the state to keep folks, keep folks healthy, I think. Any of the plagues up to the Reformation taught folks that governments have an interest in public health. (Participant #12)

### Theme 4: Community factors – many barriers to health and healthcare are present

Black communities, especially low-income Black men, experience many barriers to health and healthcare that often are one or more of the following: lack of health insurance, which is typically tied to employment; lack of time or time off from work; lack of transportation or poor public transportation; lack of trust in providers and/or institutions.

Many participants mentioned that most men in the priority population delay seeking healthcare, but if/when they do decide to seek healthcare, they must overcome several barriers. For example, one participant said, “… culturally, sometimes—not just African American men, African American people, generally—will sometimes put their health on the back burner because there’s so many other, pressing issues. They’re not wanting to engage with the healthcare system that is not necessarily friendly *[laughter]* in many ways.” (Participant #13)

In addition, health and well-being are negatively affected by several factors that are ever-present in Black communities. More than one stakeholder mentioned the lack of access to healthcare in the neighborhoods or communities where Black people typically live, along with a lack of access to healthy food options. A few participants mentioned environmental issues in Black communities (e.g., lack of investment in the built environment), frequently observed unhealthy behaviors (e.g., smoking, unhealthy eating habits, lack of physical activity), and related health issues (e.g., asthma, hypertension, diabetes). Also, a few community leaders mentioned broader issues experienced at the community level. For instance, one community leader put it this way:

… it seems within our community, a lot of times there are negatives. What’s interesting is oftentimes we don’t need to realize that the negative is attached to some historical truth. …There is that history that’s been in our community. There’s been a history of distrust in our community when it comes to police, policing. A lot of times, even though we don’t have the language for it, we just know that it’s there…We know a lot of the fears, a lot of the hesitations of people within that demographic, I would say a large majority of the time it is steeped in some type of factual truth. I think that that sometimes can be a negative because so often in our communities we have been lied to. We’ve oftentimes been left to feel like there’s nobody we can turn to, there’s nobody we can trust. I think that that’s maybe some of the bad. (Participant #11)

### Theme 5: Societal factors – racism and poverty are entrenched barriers to health

Racism and poverty, which often are intertwined, are significant barriers to the health and well-being of low-income Black men with chronic conditions. Furthermore, barriers to the health and well-being of individuals in this population are even greater for those with a history of incarceration. One community leader shared the following insight about SRD, which he referred to as systemic racism:

There’s so much research that points to how systemic racism is such a stressor for black men. …That stress is taken out oftentimes on family members. There is a high correlation between systemic racism and stress and domestic violence in African American communities. It’s not unique to African American communities, but it is prevalent. Particularly for young men who—and young women—who are at that—in the mid-30s. The lifespan where you are expected to be able to get a job and take care of your family and all those things that—the numbers are higher there. Systemic racism is a significant barrier to the physical, the psychological, the spiritual health of Black men. The consequences of that—it oftentimes is dealt with by Black women or Black partners and Black children who are most—are closer in proximity to those Black men than anybody else. That’s a significant barrier. I think it also has a significant impact on people’s—men’s ability to—their self-esteem and how they feel about what it means to be a Black man. Being a Black man who—and there is an expectation. We live in a patriarchal society, and there is an expectation, particularly in heterosexual relationships, that it doesn’t even matter those women—Black women—have been in the workforce, so to speak, since slavery. There’s still the patriarchal construct that the man brings home the bacon, so to speak. Doesn’t matter whether the woman is also bringing home the bacon. There’s still that kind of sense that I think is prevalent and an expectation that if the Black man has a family, he’s going to do this part to take care of that family. Racism across education, housing, employment, healthcare—all these systems—we don’t make this up. It’s there. The research is there. The numbers are there to show that racism has a significant impact on Black men’s mental, psychological, spiritual health. (Participant #13)

Several participants mentioned that poverty affects the health of Black men or Black communities “in every way possible” (Participant #7). When asked to identify things that may affect the health of Black men who have low incomes and may have a chronic illness, one community leader said:

The number one thing, of course, is gonna be income. Most of the issues that people have are income-related in some way or another. If they don’t have a place to live, it’s because they can’t afford it. If they don’t have food to eat, it’s because they can’t afford it. When you’re looking at Maslow’s hierarchy, all those basic needs are all income related. *[Laughter]* You know? (Participant #8)

When discussing issues like racism and poverty, a few stakeholders also talked about incarceration and its exacerbating effects on health and access to healthcare for the community. One stakeholder who was a healthcare provider characterized what he saw with his patients as “chronic toxic stress and trauma,” which he believed to be underlying reasons for the uncontrolled chronic illness that is typical among Black men with low incomes. To help explain how this showed up in a patient visit, one healthcare provider had this to say:

Yeah, I see it all the time. I really—Honestly, I see it all the time and it’s not—I see it in patients who come and are distrustful of the whole health system. By distrustful I mean just generally like, “Why should I listen to you?” “What are you going to do?” “How are you gonna help me?” “Nobody’s helped me before.” “This really doesn’t make much of a difference.” “You don’t actually care.” “You don’t know what my experience is like.” I’ve gotten a lot of that over the last several years. Even now, it’s not everybody. It’s like these little sort of bits and spurts of it, but you can feel it when you see it. Like when that patient comes in and has not had great experiences with the healthcare system—or is so fearful of their own chronic illness that they’re really closed off. When you try to engage with them, you feel that sense of mistrust. It’s really— You’re going to have to spend a lot more time just building a relationship with somebody in order for them to then bridge that gap of trust. That’s the systemic racism that—I mean it’s not—It’s definitely historical and intergenerational about perceptions around healthcare. (Participant #24)

One stakeholder who works with men who have been formerly incarcerated emphasized the need to have appropriate people who are well-suited to work with Black men who have low incomes and chronic illnesses and who may also have a history of incarceration.

I think personally, the individuals that’s working with this population must care, not just a job, must have some compassion and must care about the individual. Don’t look at ’em because they’ve been to prison, and they’re a convicted felon. Think about that person. That’s one of ’em, and the system need to put people in place that knows what they’re doin’ and not burned out, get burned out so quick. (Participant #2)

## Discussion

The primary aim of this study was to conduct a pre-implementation examination of key contextual factors, which is a foundational yet under-studied stage of intervention research. The Exploration, Preparation, Implementation, Sustainment (EPIS) Implementation Framework stresses that factors identified during the exploratory phase strongly influence the intervention’s later reach, fidelity, and outcome equity and that the fit between context and intervention is decided before intervention delivery begins [31,32]. Qualitative exploration of interest holders’ competing realities, knowledge, and perspectivesof using UBI in a future intervention to facilitate healthcare utilization and promote health equity among low-income Black men with chronic illness, including HIV, who are not engaged in healthcare is essential and cannot be specified or quantitatively measured a priori. Our findings center these perspectives and underscore a complex interplay of factors –individual, interpersonal, institutional, community, and societal – that must be addressed before (or perhaps simultaneously when) designing effective structurally-focused interventions involving the priority population. Rather than focusing on reasons why the intervention is warranted or how the intervention will be designed, our findings align with prior research on the social determinants of health and further emphasize the importance of addressing upstream structural barriers in any pre-implementation planning [33–36].

Interview participants frequently described how chronic disease management is deprioritized by the priority population when urgent concerns such as housing, employment, and family obligations dominate. This is consistent with findings from the Connecticut Men’s Health Reentry Study, which documented the difficulties formerly incarcerated men face in balancing healthcare with immediate survival needs, such as work or parole reporting requirements [37]. Contrary to stereotypes that low-income individuals are disinterested in work or reliant on assistance, participants in our study expressed that the Black men they were familiar with had a strong desire for employment, even in low-wage settings. These insights echo critiques of unfounded claims that UBI would disincentivize work [38,39]. Rather, they highlight how structural racism—manifested through wage suppression, mass incarceration and police violence, and limited access to supportive services—restricts opportunity for this population [40^,41].^

As evidenced by experiences shared in our interviews, justice-involved individuals, particularly Black men, face stark economic disadvantages post-incarceration. Within one year of release, wages among all formerly incarcerated people are approximately half that of the general population, and this wage gap persists for years, but formerly incarcerated Black and Latino people suffer greater losses in earnings over their lifetime compared to their white counterparts [42]. Unemployment among formerly incarcerated individuals remains alarmingly high, higher even than during the Great Depression [43]. However, a report released by the Bureau of Justice Statistics (2021) found that compared to all other racial and ethnic groups, white individuals were most likely to be employed after release from federal prison [44]. Higher employment and earnings among white individuals post-incarceration has been attributed by some to “racialized re-entry”, which identifies differences in social network characteristics as the reason for differential access to employment [45,46]. Though employment interventions show promise, they remain underutilized and are rarely tailored for populations facing compounded marginalization.

Layered onto these barriers are persistent racial inequities, a notion shared by multiple participants describing issues of racism and poverty. White families more frequently have intergenerational wealth or social support to buffer the financial impact of incarceration [47,48]. Respondents in our study spoke to these points, acknowledging how unfair legal and financial systems disproportionately burden some groups more than others. In the context of UBI, racial differences should continue to be examined in how groups differentially experience financial strain and how supplemental income may be more impactful for those experiencing the most dire socioeconomic disparities. As such, these disparities reinforce systemic obstacles that make consistent healthcare engagement infeasible for many Black men with chronic conditions.

The financial burdens of incarceration, which disproportionately affect Black men, also extend to families, as noted by a couple of interviewees in our study. One participant described the ongoing costs from legal fees to parole supervision to restitution. In the Southern state where this study was conducted, the average cost of a 15-minute in-state phone call from a jail coast $14.49, and the cost of a 15-minute in-state call from a state prison was $4.80 [49]. Video visitation costs are also steep, averaging $12.95 per 30-minute call [50]. Yet such contact is vital, as consistent communication with family members is associated with lower recidivism [50]. These expenses, coupled with the disruption of family life and financial instability, make health a low priority.

In this context, UBI (or guaranteed supplemental income) should not be viewed as a replacement for employment, nor a subsidy for incarceration-related fees. Instead, it should be viewed more holistically. It may serve as a stabilizing intervention that supports reentry, mitigates the stress of unmet needs, and facilitates a shift in priorities, allowing individuals to address their health conditions proactively. Evidence supporting the promise of such an intervention comes from the multiple studies conducted in different cities and with different population groups across the U.S. [22]. A modest, unconditional financial floor could help reduce the cognitive load of economic insecurity, enabling more consistent engagement with health systems.

The analysis of these interviews is our attempt at improving our understanding of the context within which our UBI intervention would take place. Critically, participants emphasized that even the best-designed interventions must be rooted in contextual conditions, trust, cultural relevance, and authenticity. As noted by interviewees, this includes providing access to providers with shared lived experience and addressing longstanding medical mistrust. Given the documented inequities in health outcomes for Black men with chronic illness, UBI represents one potential structural solution among many that must be pursued in tandem to achieve health equity, but there are factors at multiple levels of the SEM that must be addressed before any intervention is deemed feasible and acceptable by or for this population.

### Limitations

This study is not without limitations. The findings from interviews with interest holders in one southern state in the U.S. are not generalizable to the larger population, although qualitative research is not necessarily intended to be generalizable. The purpose of using qualitative methods for this study was to gain a deeper understanding of factors that need to be considered before implementing a UBI intervention to improve health and wellness among low-income Black men with a chronic health condition in a southern state. Other researchers intending to implement an intervention to improve health and/or access to healthcare for the same priority population (i.e., transferability [51]) will find our results informative and applicable to their work.

### Implications

UBI may serve as a structural lever to advance health equity by addressing the economic precarity that often undermines healthcare utilization, especially among JI individuals. Interviews revealed that financial strain – linked to incarceration, underemployment, and SRD – frequently forces people, particularly Black men, to deprioritize their health. Modest, unconditional cash transfers could reduce stress and enable health-seeking behaviors. However, barriers to care are multilayered, spanning personal fears, fragmented systems, and disinvested communities. Effective and sustainable interventions must therefore operate across the socioecological model and integrate wraparound services like transportation and legal aid. Building trust through culturally concordant providers, including Black clinicians and peer navigators with lived experience, was also seen as critical. Many participants prioritized employment over health—not from preference for aid, but due to survival imperatives and parole constraints. Financial burdens tied to incarceration further compound these challenges. Ultimately, economic stability, through policies like UBI, can reposition health as a feasible priority rather than an unaffordable luxury.

## Conclusions

Before implementing an intervention to facilitate healthcare access and promote health equity of the priority population, pre-implementation factors need to be considered at the individual, interpersonal, community, and institutional levels. Some factors that can be addressed when designing such an intervention are identifying and utilizing culturally concordant healthcare providers, building trust and rapport with members of the priority population to ensure participation, and identifying solutions for anticipated barriers to accessing healthcare (e.g., transportation). Achieving racial health equity is too complex to address in a single intervention. UBI programs are one part of a wider solution to improve health for marginalized populations such as formerly incarcerated Black men. Additional research must consider pre-implementation strategies outlined in this report to gain buy-in from interest holders and develop a sustainable intervention.
